# Automated analysis of spoken language differentiates multiple system atrophy from Parkinson’s disease

**DOI:** 10.1007/s00415-024-12828-w

**Published:** 2025-01-15

**Authors:** Martin Šubert, Tereza Tykalová, Michal Novotný, Petr Dušek, Jiří Klempíř, Jan Rusz

**Affiliations:** 1 Department of Circuit Theory, Faculty of Electrical Engineering, Czech Technical University in Prague, Technická 2, Praha 6, 16000 Prague, Czech Republic; 2 Department of Neurology and Centre of Clinical Neuroscience, First Faculty of Medicine, Charles University and General University Hospital, Prague, Czech Republic; 3 Department of Neurology and ARTORG Center, Inselspital, Bern University Hospital, University of Bern, Bern, Switzerland

**Keywords:** Multiple system atrophy, Language, Spontaneous discourse, Automated linguistic analysis, Natural language processing

## Abstract

**Background and objectives:**

Patients with synucleinopathies such as multiple system atrophy (MSA) and Parkinson’s disease (PD) frequently display speech and language abnormalities. We explore the diagnostic potential of automated linguistic analysis of natural spontaneous speech to differentiate MSA and PD.

**Methods:**

Spontaneous speech of 39 participants with MSA compared to 39 drug-naive PD and 39 healthy controls matched for age and sex was transcribed and linguistically annotated using automatic speech recognition and natural language processing. A quantitative analysis was performed using 6 lexical and syntactic and 2 acoustic features. Results were compared with human-controlled analysis to assess the robustness of the approach. Diagnostic accuracy was evaluated using sensitivity analysis.

**Results:**

Despite similar disease duration, linguistic abnormalities were generally more severe in MSA than in PD, leading to high diagnostic accuracy with an area under the curve of 0.81. Compared to controls, MSA showed decreased grammatical component usage, more repetitive phrases, shorter sentences, reduced sentence development, slower articulation rate, and increased duration of pauses, whereas PD had only shorter sentences, reduced sentence development, and longer pauses. Only slower articulation rate was distinctive for MSA while unchanged for PD relative to controls. The highest correlation was found between bulbar/pseudobulbar clinical score and sentence length (*r*** = **−0.49, *p*** = **0.002). Despite the relatively high severity of dysarthria in MSA, a strong agreement between manually and automatically computed results was achieved.

**Discussion:**

Automated linguistic analysis may offer an objective, cost-effective, and widely applicable biomarker to differentiate synucleinopathies with similar clinical manifestations.

**Supplementary Information:**

The online version contains supplementary material available at 10.1007/s00415-024-12828-w.

## Introduction

Multiple system atrophy (MSA) is a progressive neurodegenerative disorder characterized by a combination of autonomic dysfunction, parkinsonism, and cerebellar ataxia. MSA differs from Parkinson’s disease (PD) by more widespread neurodegenerative changes, resulting in additional clinical signs, more rapid disease progression, and poor response to dopamine replacement therapy [1]. However, given its similar clinical manifestations, MSA poses a significant diagnostic difficulty as it may masquerade as PD. Consequently, robust screening tools are essential, as a precise MSA diagnosis is critical in assessing prognosis, making treatment decisions, and understanding the underlying pathophysiology and associated development of new therapies [2].

The cognitive decline in MSA is faster than in PD and presents a spectrum of deficits, impacting attention, executive function, visuospatial abilities, and language processing [3, 4]. While language abnormalities reflect various cognitive perturbances [5], our knowledge of the precise character and extent of linguistic anomalies in MSA remains limited, with patients showing problems in repetition, reading, and semantic association tasks [6, 7]. Moreover, the traditional scales for neuropsychiatric assessment or specific Screening for Aphasia in NeuroDegeneration scale [8] were not sensitive enough to distinguish between language performance in MSA and PD groups [6, 7]. This may be because such language assessments are unrepresentative of everyday situations and thus might not be sufficient for advanced analyses.

Yet today, language biomarkers can be extracted from natural spontaneous speech. Such data can be analyzed by automated processing through automatic speech recognition (ASR) and natural language processing techniques. ASR employs statistical models and digital signal processing algorithms to transcribe spoken audio recordings into written text, while natural language processing utilizes advanced statistical methods to analyze sentence structure, words, and phrases for a deeper understanding of their context and meaning. However, whether automated linguistic analysis may help differentiate MSA and PD has never been investigated. Implementing a language assessment framework may face significant practical challenges due to more severe dysarthria in atypical parkinsonism, making audio-to-text transcription challenging. Therefore, the reliability of automated linguistic analysis should first be determined in clinically probable MSA patients, with the future goal of evaluating linguistic analysis as a diagnostic instrument in the very early stages of the disease.

The present study aimed to address these gaps by characterizing complex language profile in MSA patients, leveraging cutting-edge ASR and natural language processing techniques. Furthermore, we focused on determining the reliability of linguistic analysis in differentiating between MSA and PD. An additional objective was to develop and test the robustness of a fully automated linguistic analysis.

## Subjects and methods

### Standard protocol approvals, registrations, and patient consents

The study was approved by the Ethics Committee of the General University Hospital in Prague, Czechia, and has, therefore, been performed in accordance with the ethical standards laid down in the 1964 Declaration of Helsinki and its later amendments. All participants provided written, informed consent to the neurological examination and recording procedure.

### Study design and participants

Consecutive recruitment of probable MSA participants was performed from 2012 to 2020 and PD and control subjects from 2015 to 2023 at a single center of the First Medical Faculty, Charles University and General University Hospital, Prague, Czechia. All MSA patients were clinically followed for at least three subsequent years or until they died, and none of the patients exhibited red flags that would suggest a diagnosis other than MSA. PD and control groups were recruited and age- and gender-matched to MSA. A specialist in movement disorders established the diagnoses of all patients according to the consensus diagnostic criteria for MSA [9] and the Movement Disorder Society clinical diagnostic criteria for PD [10]. Disease duration was estimated based on the self-reported manifestation of the first motor symptoms. The PD patients were selected from a longitudinal project “biomarkers in PD (BIO-PD)” aimed to collect a large representative sample of de-novo PD patients [11]. The PD patients with longer reported disease duration were intentionally chosen and matched to the disease duration of MSA. Some of these PD patients had subjectively undisturbing symptoms and preferred not to be treated by dopaminergic medication at the time of diagnosis; thus, their clinical and speech data were taken from follow-up visits at year 1 or 2. At the time of examination, each treated MSA was on stable medication for at least 4 weeks, consisting of various doses of levodopa alone or combined with different dopamine agonists and/or amantadine. None of the MSA or PD patients reported a history of speech-language disorders unrelated to the manifestation of the respective neurologic disorder. A healthy control group was free of speech or motor neurologic disorder, active oncologic illness, and abuse of psychoactive substances. Each subject completed at least elementary education lasting eight years.

The clinical evaluation included structured clinical interview focused on personal and medical history, history of drug and substance use, and current medications. MSA patients were rated by the Natural History of Neuroprotection in Parkinson plus syndromes-Parkinson plus scale (NNIPPS) [12]. The NNIPPS scale was chosen as it allows the evaluation of all clinical symptoms encountered in parkinsonism in one scale, including symptoms that may potentially influence language production. PD patients were scored according to the motor subscore of Movement Disorder Society - Unified Parkinson’s Disease Rating Scale (MDS-UPDRS III) [13] and Montreal Cognitive Assessment [14, 15]. Perceptual evaluation of dysarthria severity in all groups was based on MDS-UPDRS III speech item.

### Speech assessment

All participants were guided to perform spontaneous discourse on a topic of their preference. The examination was conducted in a single session in a low-noise room, using a head-mounted condenser microphone (Beyerdynamic Opus 55, Heilbronn, Germany) positioned approximately 5 cm from the subject's mouth. The recordings utilized 48 kHz sampling and 16-bit resolution. On average, the narration was approximately 125 seconds long (standard deviation [SD] 27) and contained an average of 226 words (SD 76). The discourse's content was categorized into 7 topics: autobiographical discourse (26%), hobby (20%), career (12%), social event description (12%), holiday (10%), typical day (9%), and other topics (11%). The task duration was similar to previous studies on linguistic analysis in patients with parkinsonism [16, 17].

### Speech transcription and annotation

Speech audio data were automatically transcribed using a state-of-the-art ASR system, Whisper [18], utilizing a pre-trained model large-v2. Each audio recording resulted in a transcript with the text form of the participant’s narration, excluding paralinguistic phenomena, such as filled pauses (e.g., “ehm”, “mmm”), which are not needed for further analysis. All transcripts were analyzed using the natural language processing tool, stanza [19], and each word in the transcript was labeled with the corresponding word type necessary for linguistic analysis.

Furthermore, a manual analysis was conducted, where a speech specialist (M.Š.) transcribed each audio file and corrected annotations manually in order to evaluate the accuracy of linguistic features computed from automated analysis; the testing for accuracy of acoustic parameters has already been done previously [20, 21].

### Linguistic analysis

**Fig. 1:**
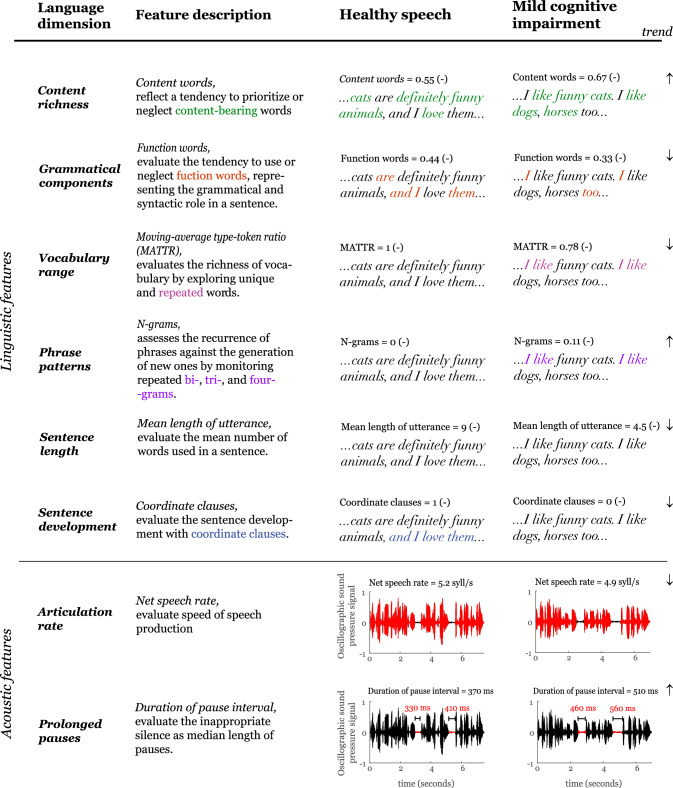
Description of linguistic and acoustic features

The feature selection process was guided by 3 primary criteria: (i) the demonstrated sensitivity to the mild cognitive impairment [16, 17], (ii) the ability to encompass multiple language domains, demanding unique computational principles and consequently resulting in minimal correlations among linguistic and acoustic variables, (iii) the feasibility of achieving fully automated analysis procedure. In line with these criteria, we have chosen 6 linguistic and 2 acoustic features (Fig. 1). Comprising a limited set of features reduces the probability of encountering a Type I error and helps to alleviate the potential for overfitting in the regression analysis.

#### Linguistic features

Content richness was assessed using *content words* [22], which reflects a potential tendency to prioritize or neglect content-bearing words, computed as the number of content words to a total number of words. Grammatical components were represented by *function words* [22], which serve grammatical and syntactic roles; thereby, the absence of function words may lead to incorrectly structured sentences. Function words are computed as the number of function words to the total number of words. Vocabulary range was evaluated with a *moving-average type–token ratio (MATTR)* [23], quantifying lexical diversity. MATTR is computed by iterating through text using a fixed window size and a step size of 1, calculating the ratio of unique words to the total number of words in each window. The final score the average of these ratios. Based on our dataset, the window size was set to 84 words. Phrase patterns were evaluated using *n-grams* [16], which count the repetition of two-, three- and four-grams, i.e., following the participant's usage of the exact phrases versus thinking of new ones, or the necessity to repeat the exact phrase multiple times before continuing in the narration. Sentence length was assessed by *mean length of utterance* [24], which counts the mean number of words in the sentence. Sentence development was assessed using *coordinate clauses* [25]*,* defined as the number of coordinate clauses normalized to the total number of clauses. We found only a moderate correlation among linguistic features (Pearson: |r|<0.48), except a strong correlation between content and function words (Pearson: *r*** = **−0.86), which was expected as these two parameters complement each other.

#### Acoustic features

The articulation rate was represented by *net speech rate* [20], assessing the speed of speech production. Net speech rate is the syllable count derived through the hyphenation method divided by the speech length, excluding pauses longer than 30 ms. Prolonged pauses were evaluated with *duration of pause interval* [21], derived as the median length of pause intervals equal to or longer than 200 ms [26]. A weak correlation was found between acoustic features (Pearson: *r*** = **−0.45).

### Statistical analysis

We employed a one-way analysis of covariance with Fisher’s least-squares difference post hoc tests, treating the group (controls vs PD vs MSA) as a between-subject factor to assess the differences. These analyses were adjusted for age, sex, and the topic of discourse as a covariate. Additionally, these analyses were adjusted for MDS-UPDRS III speech item as a covariate to eliminate the potential effect of speech impairment severity. We utilized partial correlation to examine the relationship between linguistic features and clinical data while controlling for age, sex, and the topic of discourse as a covariate; we used Shapiro-Wilk test to assess the normality of the clinical data, where Pearson partial correlation was used for normally distributed data and Spearman partial for non-normally distributed data. Additionally, Pearson correlation and root-mean square error normalized by the mean observed value (NRMSE) [27] were used to examine the relationship between linguistic features extracted from automated versus manual analysis. The accuracy of ASR was evaluated by the word error rate metric, calculated as the number of substitutions, deletions, and insertions divided by the total number of words in the reference transcript [28]. A two-tailed *p* value<0.05 was the threshold for determining statistically significant differences.

We conducted a sensitivity analysis with a binary logistic regression followed by leave-one-subject-out cross-validation to evaluate how well a set of linguistic features could differentiate between groups (i.e., sensitivity/specificity). Based on the receiver operating characteristic curve, the area under the curve (AUC) was calculated to report the diagnostic accuracy of linguistic analysis. Feature importance was determined by the absolute values of the model coefficients; the model was trained on normalized features (i.e., transformed into z scores), enabling direct comparison of resulting feature importance.

### Data availability

Individual participant data that underlie the findings of this study are available upon reasonable request from the corresponding author. The data are not publicly available due to their containing of information that could compromise the privacy of study participants.

## Results

### Participants

The MSA group comprised 39 participants (31 patients diagnosed with MSA-parkinsonian subtype and 8 patients with MSA-cerebellar subtype), the PD group comprised 39 drug-naive participants (30 patients diagnosed with tremor-dominant subtype, 7 patients with postural instability/gait difficulty subtype, and 2 patients with indeterminate subtype), and the healthy control group included 39 participants (Table 1). No between-group significance among the groups was found for age (*p*** = **0.94) and sex (*p*** = **0.59). MSA and PD also did not differ in disease duration (*p*** = **0.94).

**Table 1:** Demographic and clinical data of participants

	Healthy controls (*n*** = **39)	Parkinson’s disease (*n*** = **39)	Multiple system atrophy (*n*** = **39)	*p*-value
*General*				
Age (years)	61.6 (SD 8.1, range 43–73)	60.9 (SD 12.6, range 37–79)	61.2 (SD 7.5, range 43–73)	0.94
Male	19 (49%)	23 (59%)	19 (49%)	0.59
Disease duration	–	4.1 (SD 2.2, range 1.1–11.3)	4.2 (SD 1.6, range 1.0–7.5)	0.94
L-dopa equivalent (mg/day)	0	0	543 (SD 584, range 0–1950)	<0.001^b,c^
L-dopa usage	0	0	24 (64%)	<0.001^b,c^
Amantadine (mg/day)	0	0	104 (SD 152, range 0–600)	<0.001^b,c^
MDS-UPDRS III speech item	0.1 (SD 0.2, range 0–1)	0.6 (SD 0.5, range 0–2)	1.8 (SD 0.7, range 1–3)	<0.001^a,b,c^
MDS-UPDRS III motor score	3.2 (SD 2.9, range 0–11)	33.2 (SD 19.7, range 10–70)	–	<0.001
MoCA	26.5 (SD 1.9, range 23–30)	25.0 (SD 3.3, range 17–30)	–	0.02
*Clinical subtypes*				
MSA-P	–	–	31 (79%)	
MSA-C	–	–	8 (21%)	
TD	–	30 (77%)	–	
PIGD	–	7 (18%)	–	
Indeterminate	–	2 (5%)	–	
*NNIPPS*				
Overall score	–	–	81 (SD 31, range 35–127)	
Mental subscore	–	–	6.2 (SD 4.2, range 0–16)	
Intellectual impairment	–	–	0.7 (SD 0.7, range 0–3)	
Bradyphrenia	–	–	1.2 (SD 0.9, range 0–3)	
Loss of concentration	–	–	0.7 (SD 0.7, range 0–2)	
Bulbar subscore			7.9 (SD 3.4, range 3–15)	
Speech item (ADL)			1.7 (SD 0.9, range 0–4)	

*MDS-UPDRS*** = **Movement Disorder Society-Unified Parkinson’s Disease Rating Scale; *MoCA*** = **Montreal Cognitive Assessment; *MSA-P*** = **parkinsonian subtype of multiple system atrophy; *MSA-C*** = **cerebellar subtype of multiple system atrophy; *TD*** = **tremor-dominant; *PIGD*** = **postural instability/gait difficulty; *NNIPPS*** = **Natural History of Neuroprotection in Parkinson plus syndromes-Parkinson plus scale; *ADL*** = **activities of daily living.

^a^Significant difference between healthy controls and Parkinson’s disease.

^b^Significant difference between healthy controls and multiple system atrophy.

^c^Significant difference between Parkinson’s disease and multiple system atrophy.

### Linguistic features

**Fig. 2:**
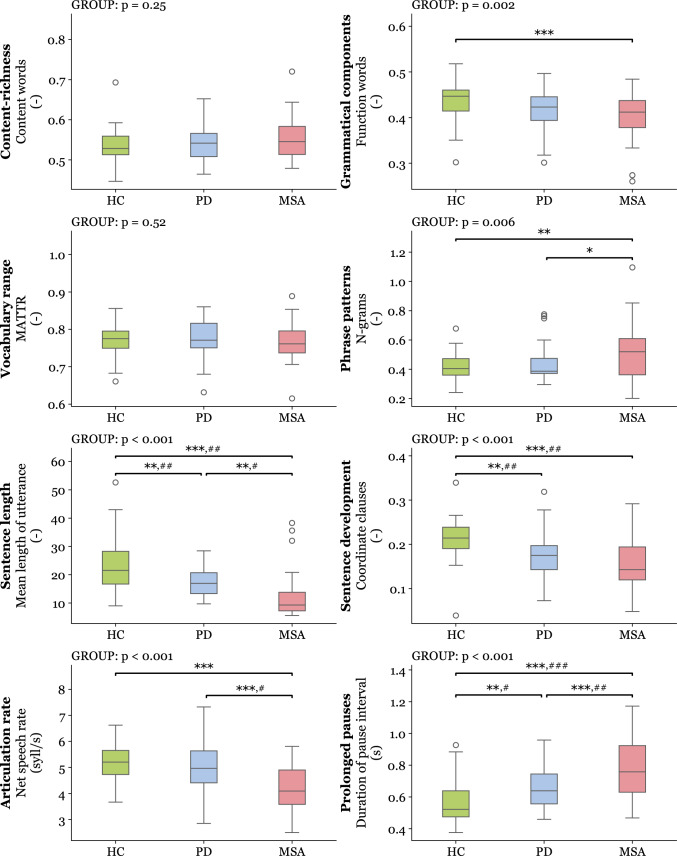
Boxplots of linguistic and acoustic features across all 3 investigated groups based on automated analysis. Horizontal lines represent the means, boxes represent 95% confidence interval, and whiskers represent the standard deviation. GROUP represent main effect after a one-way analysis of covariance. Statistically significant differences between groups after Fisher’s least-squares adjustment are shown: *p < 0.05, **p < 0.01, ***p < 0.001. All results are adjusted for the sex, age, and content of discourse. Significant difference after controlling for MDS-UPDRS III speech item as a covariate in addition to other covariates: ^#^*p*<0.05, ^##^*p*<0.05, ^###^*p*<0.001. *HC*** = **healthy controls; *PD*** = **Parkinson’s disease; *MSA*** = **multiple system atrophy; *MATTR*** = **moving-average type-token ratio

Based on automated analysis (Fig. 2), we could discriminate MSA patients from PD using phrase patterns (n-grams: *p*** = **0.016), sentence length (mean length of utterance: *p*** = **0.001), articulation rate (net speech rate: *p*<0.001), and prolonged pauses (duration of pause interval: *p*<0.001). Compared to healthy controls, MSA group was altered in grammatical components (function words: *p*<0.001), phrase patterns (n-grams: *p*** = **0.002), sentence length (mean length of utterance: *p*<0.001), sentence development (coordinate clauses: *p*<0.001), articulation rate (net speech rate: *p*<0.001), and prolonged pauses (duration of pause interval: *p*<0.001). Discrimination between PD and healthy controls was achieved with sentence length (mean length of utterance: *p*** = **0.001), sentence development (coordinate clauses: *p*** = **0.003), and prolonged pauses (duration of pause interval: *p*** = **0.009). Similar discrimination results between groups for acoustic features were obtained via a standardized reading passage (Figure S1). Correlations were found between bulbar/pseudobulbar NNIPPS score and sentence length (*r*** = **−0.49, *p*** = **0.002), and between overall NNIPPS score and articulation rate (*r*** = **−0.38, *p*** = **0.020) (Table S1). There were no significant differences between MSA patients with and without L-dopa therapy (Figure S2**, **Table S2).

### Sensitivity analysis

**Fig. 3:**
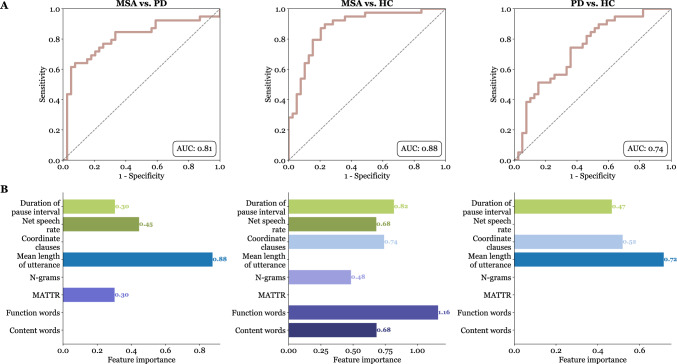
Logistic regression model evaluation and feature importance analysis. **A** Receiver operating characteristic curves with the listed AUC. **B** Linguistic and acoustic features utilized during model training, with corresponding feature importance represented as the absolute values of the model coefficients; a higher value indicates greater feature importance. *AUC*** = **area under the curve; *HC*** = **healthy controls; *PD*** = **Parkinson’s disease; *MSA*** = **multiple system atrophy; *MATTR*** = **moving-average type–token ratio

The best classification between PD and MSA groups was achieved using 2 linguistic features (mean length of utterance, and MATTR), and 2 acoustic features (net speech rate and duration of pause interval) with an AUC of 0.81 (sensitivity 77%; specificity 74%). For the classification between healthy controls and MSA groups, 4 linguistic features were utilized (content words, function words, n-grams, and coordinate clauses) and 2 acoustic features (net speech rate and duration of pause interval), resulting in an AUC of 0.88 (sensitivity 80%; specificity 85%). Between healthy controls and PD groups, the best classification was achieved using 2 linguistic features (mean length of utterance and coordinate clauses) and 1 acoustic feature (duration of pause interval) with an AUC of 0.74 (sensitivity 62%; specificity 64%) (Fig. 3).

### Comparing results from automated and manual dataset

**Fig. 4:**
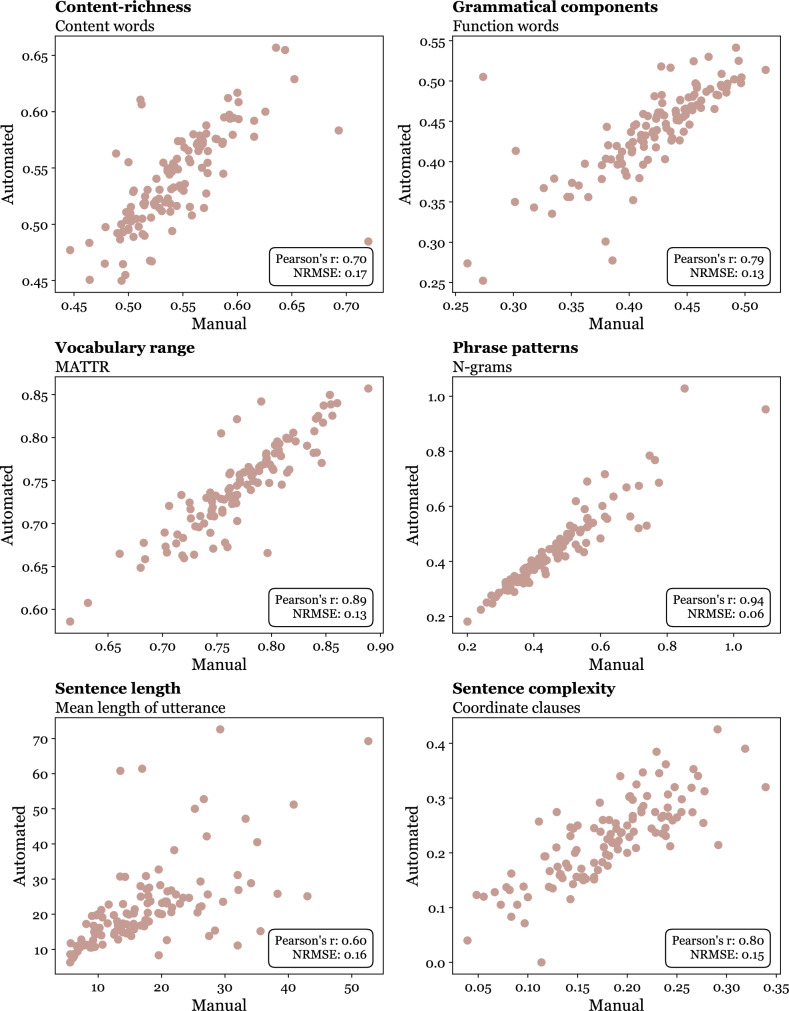
Relationship between linguistic and acoustic features extracted from manual analysis (horizontal axis) and automated analysis (vertical axis). *NRMSE*** = **normalized root-mean-square error; *Pearson r*** = **Pearson correlation coefficient; *MATTR*** = **moving-average type-token ratio

Through manual analysis, we found a strong or very strong correlation (*r*** = **0.60−0.94) and a low NRMSE <0.17 among features extracted from automated versus manual analysis (Fig. 4). We could significantly distinguish between the same groups utilizing identical features, as observed in the automated analysis (Figure S3).

Compared to the manual transcripts, the ASR system exhibited word error rates of 13.6% and 14.8% for transcripts from the healthy control and PD groups, respectively. In contrast, transcripts from the MSA group showed a notably higher word error rate of 37.9%.

## Discussion

The present study shows that automated linguistic analysis of spontaneous discourse can discriminate MSA from PD with a high AUC of 0.81. Given the strong correlation between results from manual and automated speech analysis, our findings support the possibility of employing a robust approach to detect linguistic abnormalities in parkinsonism. Such high accuracy is even more compelling with the knowledge that the present linguistic analysis is based on a monologue that lasts approximately 2 minutes with only 226 words of spontaneous speech on average. Furthermore, there is the potential for improvements in the robustness and precision of this diagnostic method through adaptation for population screening using smartphones, facilitating the collection of significantly longer audio recordings [29]. This capability can aid clinicians in making treatment recommendations and ultimately improving patient outcomes.

Language represents a complex communication system encompassing a wide range of different aspects, including grammar, vocabulary, syntactic, and lexical domains. MSA exhibited abnormalities in grammatical components, increased phrase repetitions, reduced sentence length and its development, slower articulation rate, and prolonged pauses between words. Although there are no prior studies of automated lexical and syntactic analysis in MSA, similar findings of decrease in grammatical performance, repetitions and redundant expressions, and less complex sentences have also been described in progressive supranuclear palsy [30, 31]. Therefore, these linguistic changes are rather non-specific as progressive supranuclear palsy is a tauopathy, while MSA and PD are synucleinopathies. Regarding acoustic features, MSA patients manifest slower articulation rate and extended pauses between producing the next word. The prolonged pauses likely reflect cognitive-linguistic processing, as we considered only pauses longer than 200 ms. Indeed, longer pauses have been reported in all spectrums of neurodegenerative diseases, including parkinsonism [32], mild cognitive impairment [33], and Alzheimer’s disease [26].

Despite similar disease durations, language abnormalities were generally more severe in our MSA than in PD cohort. Interestingly, contrary to our results, a previous study examined language profiles in 40 MSA patients with similar disease duration and concluded no significant difference compared to PD [7]. Therefore, we could imply that employing natural spontaneous speech tasks and extracting features representing multiple language domains could unveil more subtle impairments, which are more altered in MSA than in PD. The most feasible explanation is that the production of spontaneous discourse in MSA also largely depends on the physical ability to speak and construct longer and more complex sentences. Indeed, we found a correlation between increased bulbar severity and shorter sentences. In addition, the effect of disease progression was visible in most linguistic features, with values of the PD group intermediate between MSA patients and controls. This effect was even more pronounced when analyses were adjusted by speech impairment severity, leading to fewer linguistic differences detected between groups. Yet, we still captured differences between patients and control groups for sentence development, which cannot be interpreted as a simple effect of higher speech severity. A potential explanation behind this finding could be the presence of more cognitive abnormalities in MSA, which would align with previous research indicating a more rapid cognitive decline in MSA compared to PD [3, 4]. However, we did not observe any link between the extent of cognitive impairment and language abnormalities in MSA on mental NNIPPS subscores.

The slower articulation rate was the only feature distinctive for MSA, which was unchanged in PD relative to controls. Although the slower articulation rate in neurodegenerative diseases is typically associated with cognitive impairments [34, 35], the effect of motor orofacial movement disability on articulation rate in MSA cannot be excluded due to the role of the corticobulbar and cerebellar pathways in the development of mixed dysarthria in MSA that is not present in PD [36]. Fatigue and autonomic dysfunction such as low blood pressure, as well as breath-voice discoordination, especially in the MSA-C patients, are other factors that can limit the ability of patients to construct longer sentences, with most of them preferring to speak in short phrases with the basic words to convey the meaning. Lastly, the contribution of laryngeal innervation to a slower articulation rate cannot be excluded [37], although it needs further exploration as a slow articulation rate is common in tauopathy [38], where laryngeal pathology appears to be of a low prevalence [39]. Conversely, the speaking rate in PD can often vary and may even be faster, a phenomenon closely associated with oral festination and short "rushes" of speech [40, 41]. Indeed, self-timing impairment for motor movements might play role in increased articulation rate in PD [42, 43].

Our main findings were based on spontaneous discourse on a topic of subjects' preference, whereas standardized texts might provide relevant information on articulatory components differences in voice due to pyramidal as well as cerebellar subclinical features in MSA. However, we observed similar differences in acoustic speech patterns including slower rate and prolonged pauses between MSA and PD through standardized reading passage. The potential future advantage of linguistic analysis via spontaneous speech is that it could be robustly assessed through smartphones even in real-world scenarios [44], where it can reach better-distinguishing sensitivity due to extensive longitudinal data collected in a natural environment.

Our results indicate a high level of agreement between the features extracted using both automated and manual approaches. The differences between automated and manual labels with Pearson’s *r*>0.79 for grammatical components and phrase patterns can demonstrate the high robustness of lexical features. The high resistance against ASR error rates is attributed to the inherent nature of the features. For instance, while the ASR system may mistakenly transcribe a word like “an” as “and”, resulting in an increased word error rate, both words are function words, and thus the final score of the feature remains unaffected. Conversely, sentence length exhibited slightly higher disparities between automated and manual labels with Pearson’s *r*** = **0.60, which is attributed to the difficulty of analyzing syntactic structures. In spontaneous speech, distinguishing sentence boundaries can prove particularly challenging compared to written language.

Comparing the transcripts based on automated and manual datasets revealed a relatively high word error rate of 37.9% in the MSA group, in contrast with PD and controls, where the word error rate was lower than 15%. This is in principal agreement with a word error rate of less than 23% reported in other neurodegenerative diseases with rather mild severity of dysarthria such as multiple sclerosis [46]. In this study, MSA speech abnormalities pose a greater challenge for ASR systems due to a higher severity of dysarthria. However, our comparison with manual analysis showed that even with such a high word error rate of ASR, the linguistic analysis retains its robustness and accuracy in discrimination among investigating groups. Moreover, we can expect a lower word error rate due to the lower severity of dysarthria in earlier stages of MSA, where differential diagnosis is particularly relevant. It remains to be seen if such a word error rate would be problematic for longitudinal tracking of language changes, as measures successful in cross-sectional comparisons do not need to predict longitudinal behavior [47]. We utilized an ASR system with a general model trained on a dataset with mainly normal speech. Currently, adaptation techniques customized for impaired speech have demonstrated significant enhancements in ASR accuracy [48]. Thus, we may suggest that there is still a considerable potential to refine models using MSA speech data, thereby resulting in a notable improvement in ASR reliability.

Potential limitations of this study should be noted. For comparison to MSA, we preferred drug-naive PD patients with similar self-reported disease duration to eliminate the potential confounding beneficial effect of dopaminergic medication on language and speech [49, 50]. Our MSA cohort did not consist of de novo patients, with 62% still undergoing treatment with dopaminergic medication. While we did not detect a significant impact of L-dopa on linguistic and acoustic parameters in MSA patients, trends suggested improved performance among those receiving L-dopa, particularly in linguistic features that demonstrated the highest accuracy in differentiating PD and MSA. Therefore, as MSA patients typically exhibit greater resistance to levodopa compared to PD patients, a comparison to PD patients receiving stable dopaminergic medication would likely yield even greater diagnostic accuracy than is reported in this study. Most of our patients were diagnosed with the MSA-parkinsonian type; thus, we did not stratify between subtypes. We initially applied NNIPPS to cover all various MSA symptoms in one scale. Since common standards in PD research include MDS-UPDRS and MoCA scales, we cannot directly compare the extent of motor and cognitive impairments between our PD and MSA cohorts. On the other hand, we stratified MSA and PD patients by self-reported disease duration and speech impairment severity, methods we consider most suitable for mitigating the potential influence of disease progression on study outcomes. Since it is known that there is no significant difference in cognitive impairment between MSA and PD at early stages [6, 51], we might hypothesize that our MSA cohort matched on disease duration to PD should not exhibit substantial differences in general cognitive decline. Nevertheless, future research should perform more rigorous neuropsychological testing together with language assessment. Also, our findings are based only on the Czech language. Investigating other languages is desired to verify whether the MSA language anomalies are independent of the patient’s native language. Yet, a recent multilanguage study suggests similar cognitive-linguistic profiles of patients with parkinsonism across differing languages [17]. Moreover, the state-of-the-art ASR and natural language processing tools have been extensively developed and tailored to accommodate multiple languages worldwide without requiring extensive datasets and professional coding skills to train. Finally, we did not collect information about the length of education. However, there were sociodemographic development disadvantages for the older Czech generations, with higher education generally unavailable due to the political situation. Thus, we believe adjustment for years of education would not have made the analysis more meaningful.

In conclusion, we introduce an objective, fully automated approach based on linguistic analysis to differentiate MSA patients from PD. MSA exhibited impairments across all lexical, syntactic, and acoustic domains. Future research is required to validate and extend our approach, especially for earlier stages of the MSA. Integrating automated linguistic analysis could provide a cost-effective, time-efficient, and widely applicable novel tool to estimate the extent of language abnormalities in synucleinopathies with similar clinical manifestations.

## Supplementary Information

Below is the link to the electronic supplementary material.

Supplementary file1 (PDF 144 kb)

Supplementary file2 (PDF 506 kb)

Supplementary file3 (PDF 395 kb)

Supplementary file4 (PDF 21 kb)

Supplementary file5 (PDF 114 kb)
