# Supplementary material for: Automated analysis of spoken language differentiates multiple system atrophy from Parkinson’s disease
Source: J Neurol. 2025 Jan 15;272(2):113. doi: 10.1007/s00415-024-12828-w (PMC11735538; doi:10.1007/s00415-024-12828-w)
Supplement: Supplementary file 2 — Supplementary file2 (PDF 506 kb) [file 415_2024_12828_MOESM2_ESM.pdf]

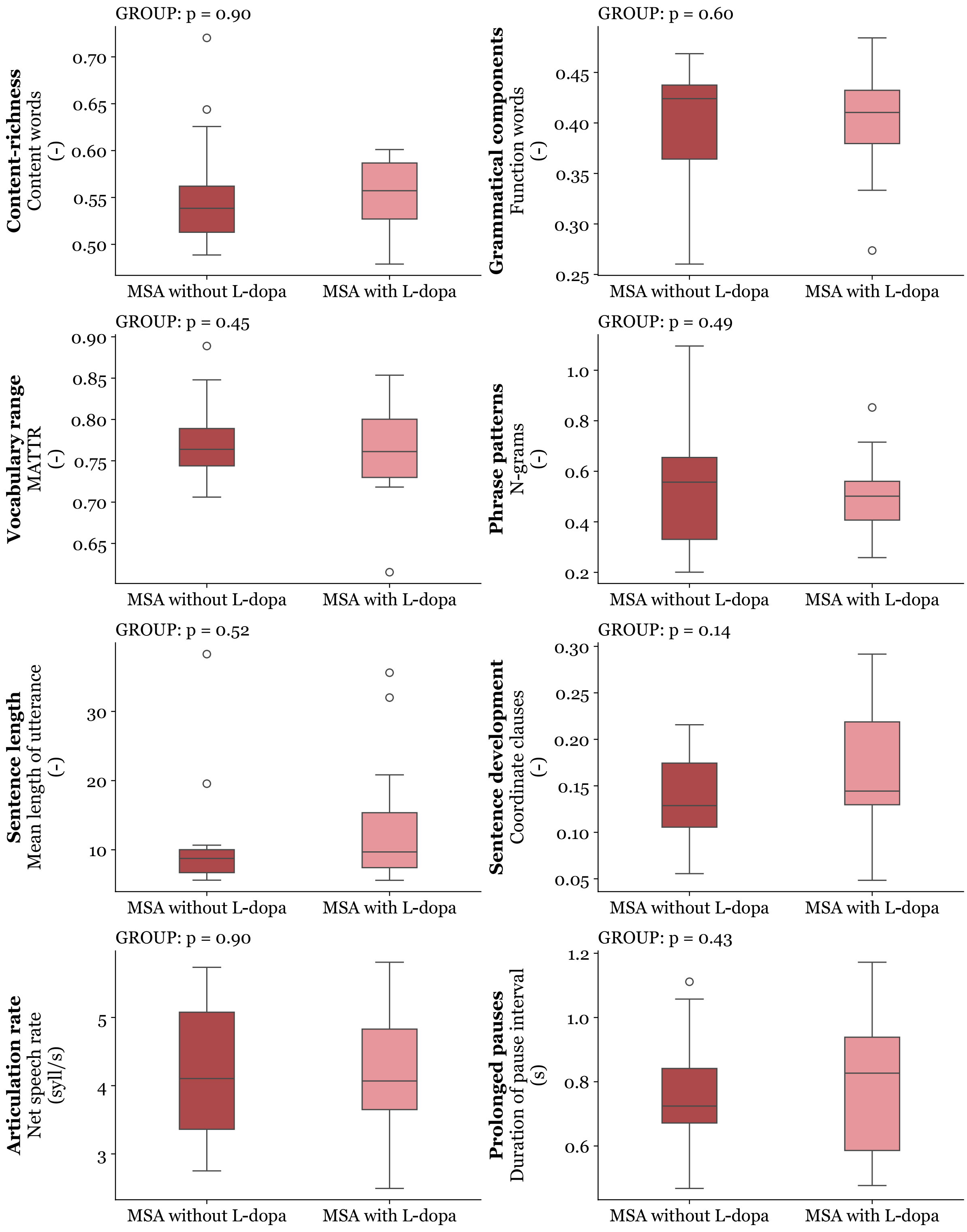

Figure S2. Boxplots of linguistic and acoustic features across MSA without L-dopa usage and MSA with L-dopa usage based on automated analysis. Horizontal lines represent the means, boxes represent 95% confidence interval, and whiskers represent the standard deviation. GROUP represent main effect after a one-way analysis of covariance. All results are adjusted for age, sex, and the content of discourse. MSA = multiple system atrophy; MATTR = moving-average type-token ratio.
