# Supplementary material for: Automated analysis of spoken language differentiates multiple system atrophy from Parkinson’s disease
Source: J Neurol. 2025 Jan 15;272(2):113. doi: 10.1007/s00415-024-12828-w (PMC11735538; doi:10.1007/s00415-024-12828-w)
Supplement: Supplementary file 3 — Supplementary file3 (PDF 395 kb) [file 415_2024_12828_MOESM3_ESM.pdf]

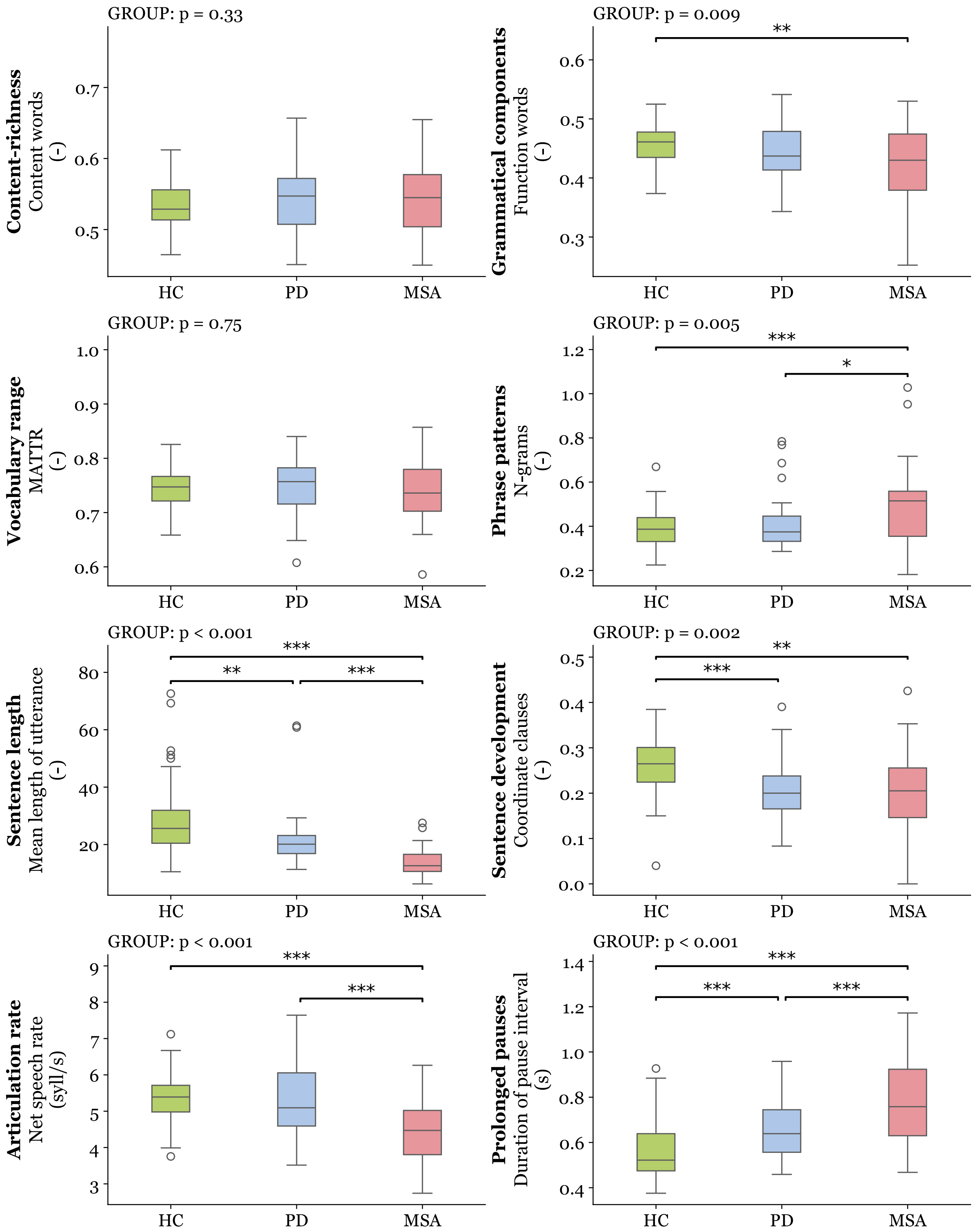

Figure S3. Boxplots of linguistic and acoustic features across all 3 investigated groups based on manual analysis. Horizontal lines represent the means, boxes represent 95% confidence interval, and whiskers represent the standard deviation. GROUP represent main effect after a one-way analysis of covariance. Statistically significant differences between groups after Fisher's least-squares adjustment are shown: *p < 0.05, **p < 0.01, ***p < 0.001. All results are adjusted for age, sex, and the content of discourse. HC = healthy controls; PD = Parkinson's disease; MSA = multiple system atrophy; MATTR = moving-average type-token ratio.
