# Supplementary material for: Automated analysis of spoken language differentiates multiple system atrophy from Parkinson’s disease
Source: J Neurol. 2025 Jan 15;272(2):113. doi: 10.1007/s00415-024-12828-w (PMC11735538; doi:10.1007/s00415-024-12828-w)
Supplement: Supplementary file 4 — Supplementary file4 (PDF 21 kb) [file 415_2024_12828_MOESM4_ESM.pdf]

Table S1. *Correlations among linguistic features and NNIPPS clinical scales.*

NNIPPS	Content richness (Content words)	Grammatical components (Function words)	Vocabulary range (MATTR)	Phrase patterns (N-grams)	Sentence length (Mean length of utterance)	Sentence complexity (Coordinate clauses)	Articulation rate (Net speech rate)	Prolonged pauses (Duration of pause interval)
Overall score[a]	0.08 (0.63)	-0.02 (0.93)	-0.18 (0.29)	0.19 (0.27)	-0.22 (0.18)	0.17 (0.32)	**-0.38 (0.020)**	-0.02 (0.89)
Mental subscore[a]	0.02 (0.90)	-0.17 (0.30)	0.20 (0.24)	0.15 (0.36)	-0.19 (0.26)	0.02 (0.91)	-0.20 (0.24)	-0.23 (0.17)
Intellectual impairment[b]	0.14 (0.41)	-0.14 (0.40)	0.15 (0.38)	-0.01 (0.95)	-0.15 (0.37)	0.07 (0.68)	-0.12 (0.47)	-0.13 (0.45)
Bradyphrenia[b]	-0.18 (0.27)	0.01 (0.96)	0.02 (0.92)	0.01 (0.97)	-0.14 (0.42)	0.10 (0.56)	-0.13 (0.45)	-0.03 (0.86)
Loss of concentration[b]	0.09 (0.58)	-0.21 (0.22)	0.22 (0.19)	-0.06 (0.72)	-0.15 (0.39)	-0.13 (0.43)	-0.04 (0.80)	-0.26 (0.13)
Bulbar/pseudobulbar subscore[a]	-0.04 (0.80)	-0.03 (0.85)	0.18 (0.30)	0.28 (0.10)	**-0.49 (0.002)**	-0.14 (0.42)	-0.31 (0.061)	-0.05 (0.76)
Speech item (motor examination)[b]	-0.05 (0.76)	-0.05 (0.77)	-0.02 (0.89)	0.27 (0.10)	-0.28 (0.094)	0.14 (0.41)	-0.24 (0.15)	-0.01 (0.96)
Speech item (ADL)[b]	-0.00 (0.98)	-0.03 (0.86)	-0.18 (0.28)	0.13 (0.46)	**-0.41 (0.011)**	0.18 (0.28)	**-0.41 (0.013)**	-0.06 (0.72)

Data are the partial correlation *r* (*p*-value). MATTR = moving-average type-token ratio; NNIPPS = Natural History of Neuroprotection in Parkinson plus syndromes-Parkinson plus scale; ADL = activities of daily living.
[a]Pearson partial correlation.
[b]Spearman partial correlation.
