# Supplementary material for: Automated analysis of spoken language differentiates multiple system atrophy from Parkinson’s disease
Source: J Neurol. 2025 Jan 15;272(2):113. doi: 10.1007/s00415-024-12828-w (PMC11735538; doi:10.1007/s00415-024-12828-w)
Supplement: Supplementary file 5 — Supplementary file5 (PDF 114 kb) [file 415_2024_12828_MOESM5_ESM.pdf]

Table S2. *Demographic and clinical data of multiple system atrophy participants stratified by L-dopa usage.*

	MSA without L-dopa (n=15, 38%)	MSA with L-dopa (n=24, 62%)	*p*-value
General			
Age (years)	61.8 (SD 7.6, range 43-72)	60.8 (SD 7.7, range 45-73)	0.71
Male	8 (53%)	11 (46%)	0.66
Disease duration	4.2 (SD 1.9, range 1.0–7.5)	4.1 (SD 1.4, range 2.0-7.0)	0.97
MDS-UPDRS III speech item	1.9 (SD 0.9, range 1-3)	1.8 (SD 0.6, range 1-3)	0.58
NNIPPS			
Overall score	85 (SD 39, range 35-127)	80 (SD 25, range 41-125)	0.70
Mental subscore	7.0 (SD 4.5, range 0-14)	5.8 (SD 4.1, range 0-16)	0.44
Intellectual impairment	0.6 (SD 0.6, range 0-2)	0.8 (SD 0.8, range 0-3)	0.35
Bradyphrenia	1.4 (SD 1.1, range 0-3)	1.2 (SD 0.8, range 0-3)	0.48
Loss of concentration	0.8 (SD 0.7, range 0-2)	0.6 (SD 0.7, range 0-2)	0.64
Bulbar/pseudobulbar subscore	9.2 (SD 4.2, range 3-14)	7.1 (SD 2.8, range 3-15)	0.07
Speech item (ADL)	1.8 (SD 1.0, range 0-3)	1.6 (SD 0.8, range 1-4)	0.50

MSA = Multiple system atrophy; MDS-UPDRS = Movement Disorder Society-Unified Parkinson's Disease Rating Scale; NNIPPS = Natural History of Neuroprotection in Parkinson plus syndromes-Parkinson plus scale; ADL = activities of daily living.
